# The location of the bladder neck in postoperative cystography predicts continence convalescence after radical prostatectomy

**DOI:** 10.1186/s12894-018-0370-3

**Published:** 2018-05-30

**Authors:** Susumu Kageyama, Tetsuya Yoshida, Masayuki Nagasawa, Shigehisa Kubota, Keiji Tomita, Kenichi Kobayashi, Ryosuke Murai, Teruhiko Tsuru, Eiki Hanada, Kazuyoshi Johnin, Mitsuhiro Narita, Akihiro Kawauchi

**Affiliations:** Department of Urology, Shiga University of Medical Science, Seta Tsukinowa-cho, Otsu, Shiga, 520-2192 Japan

**Keywords:** Bladder neck location, Radical prostatectomy, Continence recovery

## Abstract

**Background:**

This study was conducted to determine whether the location of the bladder neck in postoperative cystography predicts recovery of continence after radical prostatectomy.

**Methods:**

Between 2008 and 2015, 203 patients who underwent laparoscopic radical prostatectomy (LRP, *n* = 99) and robot assisted radical prostatectomy (RARP, *n* = 104) were analyzed. The location of the bladder neck was visualized by postoperative routine cystography, and quantitative evaluation of the bladder neck position was performed according to the bladder neck to pubic symphysis (BNPS) ratio proposed by Olgin et al. (J Endourol, 2014). Recovery of continence was defined as no pad use or one security pad per day. To determine the predictive factors for recovery of continence at 1, 3, 6 and 12 months, several parameters were analyzed using logistic regression analysis, including age (≤68 vs. > 68, BMI (≤23.4 vs. > 23.4 kg/m^2^), surgical procedure (LRP vs. RARP), prostate volume (≤38 vs. > 38 mL), nerve-sparing technique, vesico-urethral anastomosis leakage, and BNPS ratio (≤0.59 vs. > 0.59).

**Results:**

The mean postoperative follow-up was 1131 days (79–2880). At 1, 3, 6 and 12 months after surgery, continence recovery rates were 25, 53, 68 and 81%, respectively. Although older age (> 68) and RARP were significant risk factors for incontinence within 3 months, neither was significant after 6 months. A high BNPS ratio (> 0.59) was the only significant risk factor for the persistence of incontinence at all observation points, up to 12 months.

**Conclusions:**

A lower bladder neck position after prostatectomy predicts prolonged incontinence.

**Electronic supplementary material:**

The online version of this article (10.1186/s12894-018-0370-3) contains supplementary material, which is available to authorized users.

## Background

Rates of detection of localized prostate cancer have increased with early detection using serum PSA screening and this in turn has led to increased numbers of radical prostatectomies. Postoperative urinary incontinence may occur in a proportion of patients who undergo radical prostatectomy and is one of the most serious complications impacting the quality of life of patients. Several clinical characteristics have been reported to be critical factors predicting postoperative continence recovery, including the patient’s age [1–3], BMI [2, 4], prostate size [2], nerve-sparing technique [5], vesico-urethral anastomosis leakage [6], and so on. Various procedures have been reported to prevent postoperative incontinence, however, surgeons have not been able to overcome this complication completely [7].

In some institutions, postoperative cystography is performed routinely before removing the Foley catheter to confirm no vesico-urethral anastomosis leakage. Several physicians have reported that the postoperative cystogram findings predict continence after radical prostatectomy [8–12]. In their prostatectomy series, Jeong et al. reported that the vesico-urethral anastomosis location (VUAL) visualized by routine postoperative cystography correlated with early recovery of postoperative continence [9]. They showed a higher location of the bladder neck was correlated with better recovery of continence. Olgin et al. also reported a similar finding, using their original quantitative evaluation of the bladder neck position in a routine cystography after RARP [10].

In this study we investigated whether the location of the bladder neck in postoperative cystography in our LRP and RARP series predicted recovery of continence within 12 months of surgery.

## Methods

Two hundred and three patients who underwent LRP (*n* = 99) and RARP (*n* = 104) for clinically localized prostate cancer in the Shiga University of Medical Science Hospital from 2008 to 2015 were evaluated. All clinical, pathological and radiographical data were collected from medical records. The patients’ demographics are presented in Table 1. This retrospective observational study was approved by the internal ethical committee of Shiga University of Medical Science.

**Table 1:** Patients’ characteristics and operative data

Mean age (range)	67.7 (48–76)
Mean BMI (kg/m2), (range)	23.7 (16.6–33.2)
Mean preoperative PSA (ng/mL), (range)	9.21 (2.24–43.20)
Clinical T stage, (%)	
T1b	3 (1.5)
T1c	122 (60.1)
T2a	45 (22.1)
T2b	15 (7.4)
T2c	18 (8.9)
D’Amico Risk Criteria, (%)	
Low risk	40 (19.7)
Intermediate risk	108 (53.2)
High risk	55 (27.1)
Operation procedure, (%)	
Laparoscopic	99 (48.8)
Robot-assisted	104 (51.2)
Nerve-sparing, (%)	
Bilateral	24 (11.8)
Unilateral	61 (30.0)
Non-sparing	118 (58.1)
Mean operative time (min), (range)	257 (137–612)
Mean estimated blood loss (g), (range)	378 (10–2431)
Mean prostate volume (mL), (range)	41 (18–92)
Positive surgical margin, (%)	56 (27.5)
Vesicourethral anastomosis leakage, (%)	16 (7.9)
Mean Postoperative followup (days)	1131 (79–2880)

The surgical procedure briefly is as follows: The Retzius space was approached extraperitoneally (LRP) or transperitoneally (RARP) and an antegrade radical prostatectomy was performed with bilateral pelvic lymph node dissection. A nerve-sparing technique was indicated individually according to various conditions, including PSA value, Gleason score, number and location of positive cores and the patient’s desire to preserve sexual function. Bilateral and unilateral nerve-sparing techniques were performed in 10 (12%) and 24 (30%) cases, respectively. The vesico-uretharal anastomosis was performed with a Van Velthoven running suture. All patients underwent posterior and anterior reconstructions.

**Fig. 1:**
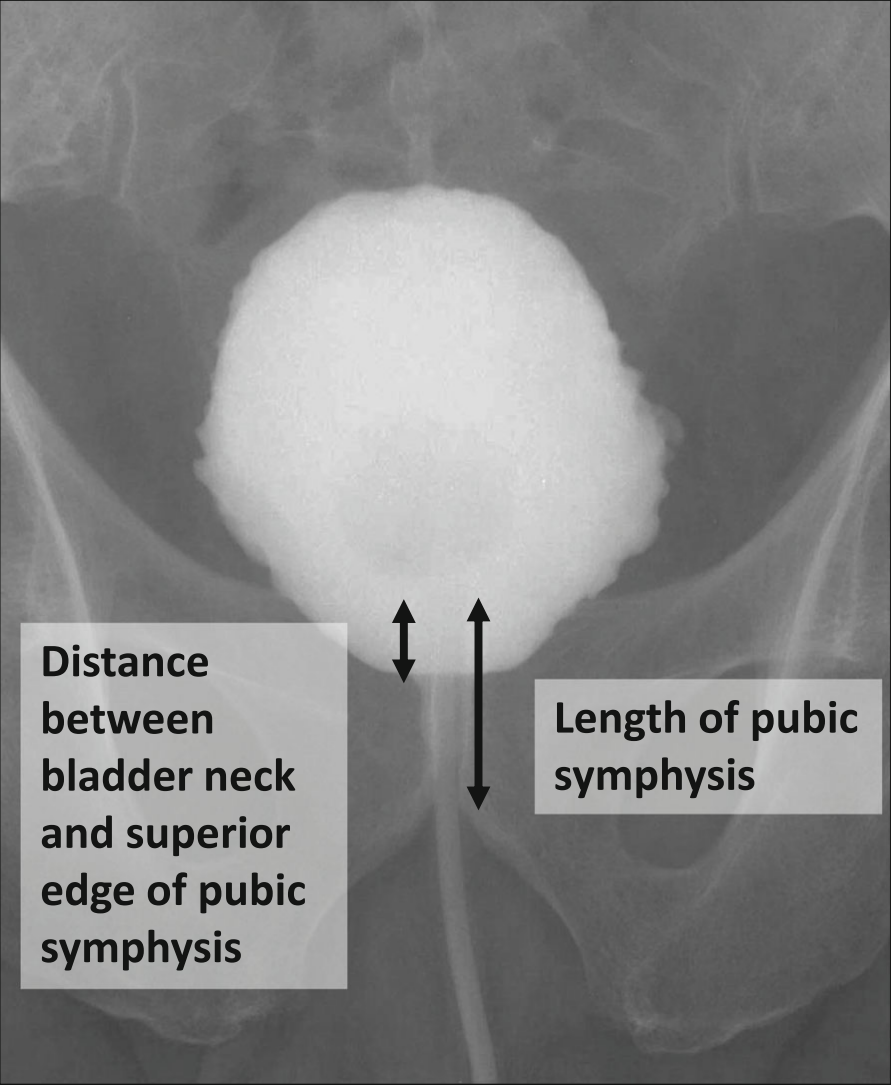
Representative cystogram after surgery

Cystography was carried out routinely six days after surgery. Images were obtained in the supine position without abdominal straining and the bladder was filled with ~ 100 mL of diluted contrast medium. A Foley catheter was advanced a few centimeters into the bladder in order to visualize the bladder neck clearly. When vesico-urethral anastomosis leakage was observed, catheter removal was delayed and a repeat cystography was performed every week until the leakage resolved. To evaluate the position of the bladder neck quantitatively, we calculated the bladder neck to pubic symphysis (BNPS) ratio, as proposed by Olgin et al. [10]. The BNPS ratio was calculated by measuring the distance from the superior edge of the pubic symphysis to the bladder neck and dividing this by the total pubic symphysis length in the cystogram. A representative cystogram is shown in Fig. 1. A single physician (SKa), who was blinded to the clinical data of the patients, evaluated cystograms and determined the BNPS ratio. Recovery of continence was defined as wearing no pad or using one safety pad per day.

Univariate analyses were performed using student t-test or the chi-square test. Uni- and multivariate logistic regression analyses were also performed. When *p*-values were less than 0.25 in univariate logistic regression analysis, the variable was included in the next multivariate logistic regression analysis. These statistical analyses were carried out using IBM SPSS Statistics version 22 software (IBM Japan, Tokyo, Japan). A *P*-value less than 0.05 is considered statistically significant.

## Results

**Fig. 2:**
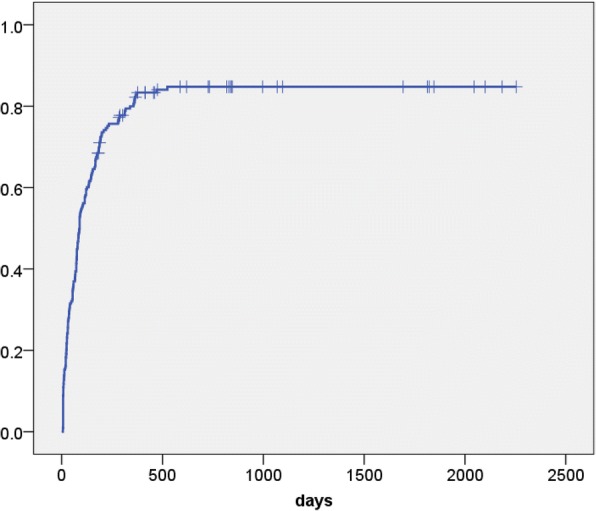
Cumulative continence recovery curve of all patients (*n* = 203)

**Fig. 3:**
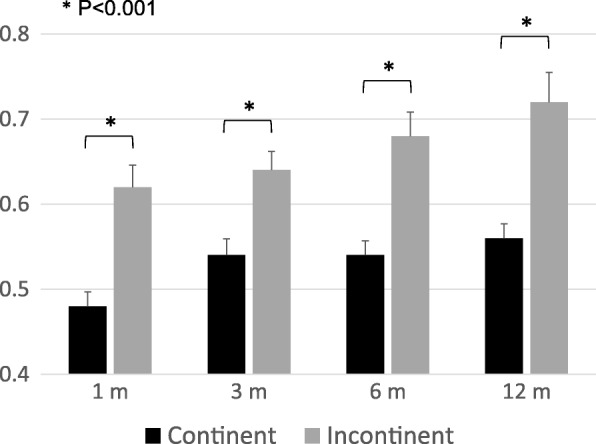
Mean BNPS ratio of the continent and incontinent groups at 1, 3, 6 and 12 months after surgery

The mean postoperative follow-up period was 1131 days (79–2880). Vesico-urethral anastomosis leakage was observed in 16 cases (8%) and catheter removal was delayed in these patients. Thereafter, repeat cystography was performed every week until the leakage resolved. The mean catheter replacement period was 19.7 days (11–36) in the patients with anastomosis leakage. A cumulative continence recovery curve of all patients is presented in Fig. 2. The continence status at 1, 3, 6 and 12 months after surgery was evaluated in an interview by the physician during regular follow-up visits and the numbers of evaluable patients were 203 (100%), 202 (99%), 190 (93%) and 171 (84%), respectively. Continence recovery rates at 1, 3, 6 and 12 months were 25, 53, 68 and 81%, respectively. At each evaluation point, several clinical parameters, including the patient’s age, surgical procedure, BMI, initial PSA value, D’Amico risk criteria, nerve-sparing procedure, operative time, estimated blood loss, prostate volume, positive surgical margin, anastomosis leakage and BNPS ratio were compared between continent and incontinent patients (Table 2 and Additional file 1: Tables S1-S4). At 1 month after surgery, the patient’s age, operation procedure and BNPS ratio differed significantly between the continent and incontinent groups. At 3 and 6 months, the patient’s age and BNPS ratio were significantly different. However, at 12 months, only the BNPS ratio was differed significantly between the groups. The difference of mean BNPS ratio between the continent and incontinent groups was significant at all evaluation points (Fig. 3).

**Table 2:** Results of statistical analysis of perioperative characteristics between continent and incontinent patients at 1, 3, 6 and 12 months

Follow-up	1 m	3 m	6 m	12 m
Continent (n)	51	107	130	139
Incontinent (n)	152	95	60	32
	*P*-Value			
Age	0.0020	0.0056	0.0031	0.0512
Procedure	0.0010	0.6601	0.9215	0.3073
BMI	0.5920	0.7470	0.6147	0.8844
PSA	0.3154	0.8596	0.2856	0.7107
D’Amico risk criteria	0.1437	0.4865	0.2614	0.0919
Nerve-sparing	0.2318	0.4737	0.4744	0.3259
Operative time	0.8303	0.7510	0.4918	0.5753
Estimated Blood loss	0.3971	0.2866	0.2039	0.4344
Prostate volume	0.4027	0.5929	0.3245	0.3158
Positive resection margin	0.9801	0.8345	0.3967	0.8450
Anastomosis leakage	0.9906	0.4413	0.1902	0.1285
BNPS ratio	< 0.0001	0.0006	< 0.0001	0.0003

Next, in order to elucidate the risk factors for delayed recovery of continence, we evaluated our patients’ data by logistic regression analysis. According to the risk factors reported previously in the literature, we chose several variables for analysis, including the patient’s age, BMI, nerve-sparing technique, prostate volume, vesico-urethral anastomosis leakage and BNPS ratio. In addition, we included the surgical procedure, because this showed a significant difference at one month in our cohort. Continuous variables, including age, BMI, prostate volume and BNPS ratio, were divided into two categories according to the median values. The median values of age, BMI, prostate volume and BNPS ratio were 68y, 23.4 kg/m^2^, 38 mL and 0.59, respectively. In order to determine whether other clinical factors were confounding the BNPS ratio, we compared these factors between the high (> 0.59) and low (≤0.59) BNPS ratio groups (Additional file 1: Table S5). The BNPS ratio did not correlate with any other factors.

At one month, the univariate logistic regression analysis showed that the older age, RARP and higher BNPS ratio correlated with persistent incontinence (Table 3). Similarly, a multivariate logistic regression analysis showed that older age (OR 2.171, 95% CI 1.011–4.663), RARP (OR 3.131, 95% CI 1.528–6.417) and higher BNPS ratio (OR 2.867, 95% CI 1.49–5.831) were significant risk factors. At three months, the univariate logistic regression analysis showed that the older age and higher BNPS ratio correlated significantly with prolonged recovery of continence. Multivariate logistic regression analysis showed that older age (OR 2.009, 95% CI 1.127–3.581) and higher BNPS ratio (OR 2.245, 95% CI 1.265–3.983) were significant independent variables. However, at 6 and 12 months, only the BNPS ratio was a significant predictor in uni- and multivariate logistic regression analyses. Therefore, the BNPS ratio was the only constant predictor, not only in short-term but also long-term continence recovery within a year of prostatectomy.

**Table 3:** Uni- and multivariate logistic regression analysis of the parameters for incontinence status at 1, 3, 6 and 12 months

Follow-up	1 m	3 m	6 m	12 m	1 m	3 m	6 m	12 m
	Univariate logistic regression				Multivariate logistic regression			
Parameters	OR (95% CI) *P*-value				OR (95% CI) *P*-value			
Age	2.442	2.102	1.826	1.527	2.171	2.009	1.690	–
(< 68 vs. > 68)	(1.222–4.881)	(1.193–3.704)	(0.983–3.392)	(0.706–3.305)	(1.011–4.663)	(1.127–3.581)	(0.898–3.180)	
	*P* = 0.011	*P* = 0.010	*P* = 0.057	*P* = 0.282	*P* = 0.047	*P* = 0.018	*P* = 0.104	
Procedure	3.008	1.132	1.031	1.492	3.131	–	–	–
(LRP vs. RARP)	(1.534–5.898)	(0.651–1.968)	(0.559–1.901)	(0.690–3.227)	(1.528–6.417)			
	*P* = 0.001	*P* = 0.660	*P* = 0.921	*P* = 0.309	*P* = 0.002			
BMI	0.843	1.041	0.798	0.483	–	–	–	0.449
(< 23.4 vs. > 23.4 kg/m2)	(0.447–1.592)	(0.599–1.808)	(0.432–1.473)	(0.219–1.064)				(0.198–1.017)
	*P* = 0.599	*P* = 0.888	*P* = 0.470	*P* = 0.071				*P* = 0.055
Nerve-sparing	1.474	1.228	1.256	1.493	1.323	–	–	–
(Yes vs. No)	(0.779–2.791)	(0.700–2.154)	(0.672–2.347)	(0.669–3.332)	(0.634–2.763)			
	*P* = 0.233	*P* = 0.474	*P* = 0.475	*P* = 0.328	*P* = 0.456			
Prostate volume	1.040	0.921	1.143	0.895	–	–	–	–
(< 38 vs. > 38 mL)	(0.551–1.961)	(0.530–1.601)	(0.619–2.109)	(0.415–1.933)				
	*P* = 0.904	*P* = 0.772	*P* = 0.669	*P* = 0.778				
Anastomosis leakage	1.007	1.495	2.014	2.389	–	–	1.817(0.609–5.426)	2.747(0.820–9.198)
(No vs. Yes)	(0.310–3.274)	(0.534–4.183)	(0.695–5.839)	(0.756–7.551)				
	*P* = 0.991	*P* = 0.444	*P* = 0.197	*P* = 0.138			*P* = 0.285	*P* = 0.101
BNPS ratio	3.127	2.333	2.060	2.371	2.867	2.245	1.989	2.338
(< 0.59 vs. > 0.59)	(1.581–6.188)	(1.326–4.106)	(1.103–3.850)	(1.063–5.290)	(1.409–5.831)	(1.265–3.983)	(1.056–3.745)	(1.034–5.288)
	*P* = 0.001	*P* = 0.003	*P* = 0.023	*P* = 0.035	*P* = 0.004	*P* = 0.006	*P* = 0.033	*P* = 0.041

## Discussion

Urinary incontinence is a frequent negative outcome of radical prostatectomy and postoperative incontinence rates of 8~ 20% have been reported in large series [13, 14]. For patients, recovery of urinary continence is one of the most important concerns with respect to quality of life [15]. Several predictive preoperative characteristics have been reported to correlate with recovery of continence [1, 2, 7]. In particular, older age, obesity and large prostate volume have been reported as the worst predictors of continence convalescence. The preoperative urethral length, measured by magnetic resonance imaging, was suggested to be a prognostic factor [16, 17]. Intra- or postoperative factors were also analyzed as predictors of post-prostatectomy incontinence and were as follows: nerve-sparing techniques, posterior/anterior reconstruction, bladder neck preservation, periurethral suspension, pelvic floor muscle exercise, incontinence volume at a very early phase after catheter withdrawal, and so on [7].

Postoperative cystography findings also were reported as a predictive factor for continence recovery by a few groups [9–12]. Jeong et al. reported a correlation between VUAL and early continence recovery in a large cohort [9]. They categorized their 678 cases into three groups, based on the vesico-urethral anastomosis location (VUAL), as determined by postoperative routine cystography: group I - above the upper margin of the symphysis pubis, group II – between the upper margin and the middle of the symphysis pubis, and group III – below the middle of the symphysis pubis. Group I showed the best recovery rate, while group III had the worst continence convalescence. They concluded that a higher VUAL leads to a higher rate of early continence recovery. In their study, they mentioned that the critical point of VUAL was the middle of the symphysis pubis. Olgin and colleagues also reported that the higher bladder neck position correlated with good continence recovery after RARP [10]. They devised a new quantitation parameter, the BNPS ratio, which was calculated on the basis of a postoperative cystogram. The BNPS ratios derived from the postoperative cystograms of 215 patients who underwent RARP were evaluated and compared with their continence status. At three months after surgery, continent patients had a mean BNPS ratio of 0.39, while incontinent patients had a mean BNPS ratio of 0.49 (*p* = 0.01). At 12 months, the mean BNPS ratio was 0.40 for continent patients, whereas incontinent patients had a mean BNPS ratio of 0.60 (*p* = 0.001). The authors concluded that the BNPS ratio on cystogram correlates with continence rates and the lower position of the bladder neck may predict a risk for prolonged incontinence. These two studies and the data presented here confirm that the correct suspension of the bladder neck predicts good recovery of continence and the critical position is around the middle of the symphysis pubis on a postoperative cystogram.

What contributes to achieving a higher bladder neck position after prostatectomy? Extensive studies of pelvic anatomy have been carried out and much effort has been devoted to improving surgical techniques. A common concept pervading these procedures is the conservation of the original anatomical structure. The restoration of the posterior rhabdosphincter, reported by Rocco et al., was a landmark in the progress of surgical technique [18, 19]. The authors emphasized that reconstruction of the posterior musclofascial plate and suspension of the urethral sphincteric complex is the key to the early recovery of continence. They reported that their procedure with posterior reconstruction dramatically shortened recovery periods (72, 79 and 86% at 3, 30 and 90 days compared with 14, 30 and 46%, respectively, without posterior reconstruction) [18]. Another pivotal modification is the anterior reconstruction. Various procedures were reported by a number of surgeons, including periurethral suspension stitch [20], preservation of puboprostatic collar [21] and puboprostatic ligament preservation [22]. Later, some authors reported on the importance of the total reconstruction procedure, which includes both anterior and posterior reconstructions. Tewari et al. presented the results of a prospective study to compare continence recovery rates of no reconstruction, anterior reconstruction only and total reconstruction [8]. They reported that total reconstruction enabled a statistically significant early return to continence (38, 83, 91, and 97% at 1, 6, 12, and 24 weeks, respectively) compared with no reconstructive procedure (13, 35, 50, and 62%) or with only anterior reconstruction (27, 59, 77, and 86%). They also presented typical cystograms of these three groups and the bladders of the total reconstruction group showed the highest vesico-urethral junctions [8]. Therefore, the suspension and stabilization of the bladder neck by anatomical reconstruction was believed to contribute to an early return of continence. All of our cases were performed with both posterior and anterior reconstructions. Consequently, it was not possible to differentiate these cases from patients without the use of these techniques. A lower bladder neck position after surgery might reflect unsuccessful anatomical preservation.

After prostatectomy, most patients achieve final continence status within 1 year; therefore, we choose the postoperative observation points of 1, 3, 6 and 12 months for our cases. Interestingly, the mean BNPS ratios of both the continent and incontinent groups increased gradually over the postoperative period (Fig. 3). Olgin et al. evaluated their patients at 3 and 12 months and reported that the mean BNPS ratios of the incontinence group were 0.49 and 0.60, respectively. Similar to our result, the BNPS ratio at 12 months was higher than at 3 months. Taking their and our findings into account, patients with a BNPS ratio of 0.6 or more might constitute the poorest group in terms of postoperative incontinence. Naturally, the postoperative bladder neck position cannot constitute a predictive factor before prostatectomy, therefore, the BNPS ratio is not valuable as a preoperative predictor of incontinence. However, the BNPS ratio can be useful as a prognostic factor which may be obtained easily by a routine postoperative cystogram and may be useful, for example, to identify patients who require strict instructions for postoperative recovery of continence, such as pelvic floor muscle exercise. For surgeons, the BNPS ratio might be used as a surrogate predictor for the degree of achievement of preservation of anatomical support of the bladder neck/sphincter complex. Further research on this point is needed.

Our study has some limitations. First, our data were obtained from the experience at a single institution and the number of patient is relatively small. Second, we evaluated the position of the bladder neck in a restricted condition, in that cystography was performed in the supine position without straining or standing. With increased abdominal pressure, the bladder neck presumably will present in a lower position. Third, we did not evaluate various other parameters which may correlate with incontinence. Pre- and/or post-operative membranous urethral length, preoperative LUTS, preoperative incontinence, and the surgeon’s extent of experience were not evaluated in our series, because of a lack of adequate data. However, regardless of these limitations, the location of the bladder neck visualized by a cystogram may become a helpful predictor of post-prostatectomy incontinence.

## Conclusion

A lower location of the bladder neck in postoperative routine cystography predicts incontinence, not only in the short term but also in the long term. According to previous reports of reconstruction techniques, it is assumed that stabilization and suspension of the urethral sphincteric complex by total reconstruction is an important procedure to achieve a higher bladder neck position.

## Additional file

Additional file 1: **Table S1**. Perioperative characteristics of the continent and incontinent patients at 1 month. **Table S2**. Perioperative characteristics of the continent and incontinent patients at 3 months. **Table S3**. Perioperative characteristics of the continent and incontinent patients at 6 months. **Table S4**. Perioperative characteristics of the continent and incontinent patients at 12 months. **Table S5**.Comparison of perioperative clinical factors between the low- and high-BNPS ratio groups. (PPTX 51 kb)

## Abbreviations

LRP: Laparoscopic radical nephrectomy
PSA: Prostate specific antigen
RARP: Robot assisted radical prostatectomy
