# Supplementary material for: The location of the bladder neck in postoperative cystography predicts continence convalescence after radical prostatectomy
Source: BMC Urol. 2018 May 30;18:52. doi: 10.1186/s12894-018-0370-3 (PMC5977542; doi:10.1186/s12894-018-0370-3)
Supplement: Supplementary file 1 — Table S1. Perioperative characteristics of the continent and incontinent patients at 1 month. Table S2. Perioperative characteristics of the continent and incontinent patients at 3 months. Table S3. Perioperative characteristics of the continent and incontinent patients at 6 months. Table S4. Perioperative characteristics of the continent and incontinent patients at 12 months. Table S5.Comparison of perioperative clinical factors between the low- and high-BNPS ratio groups. (PPTX 51 kb) [file 12894_2018_370_MOESM1_ESM.pptx]

## Slide 1
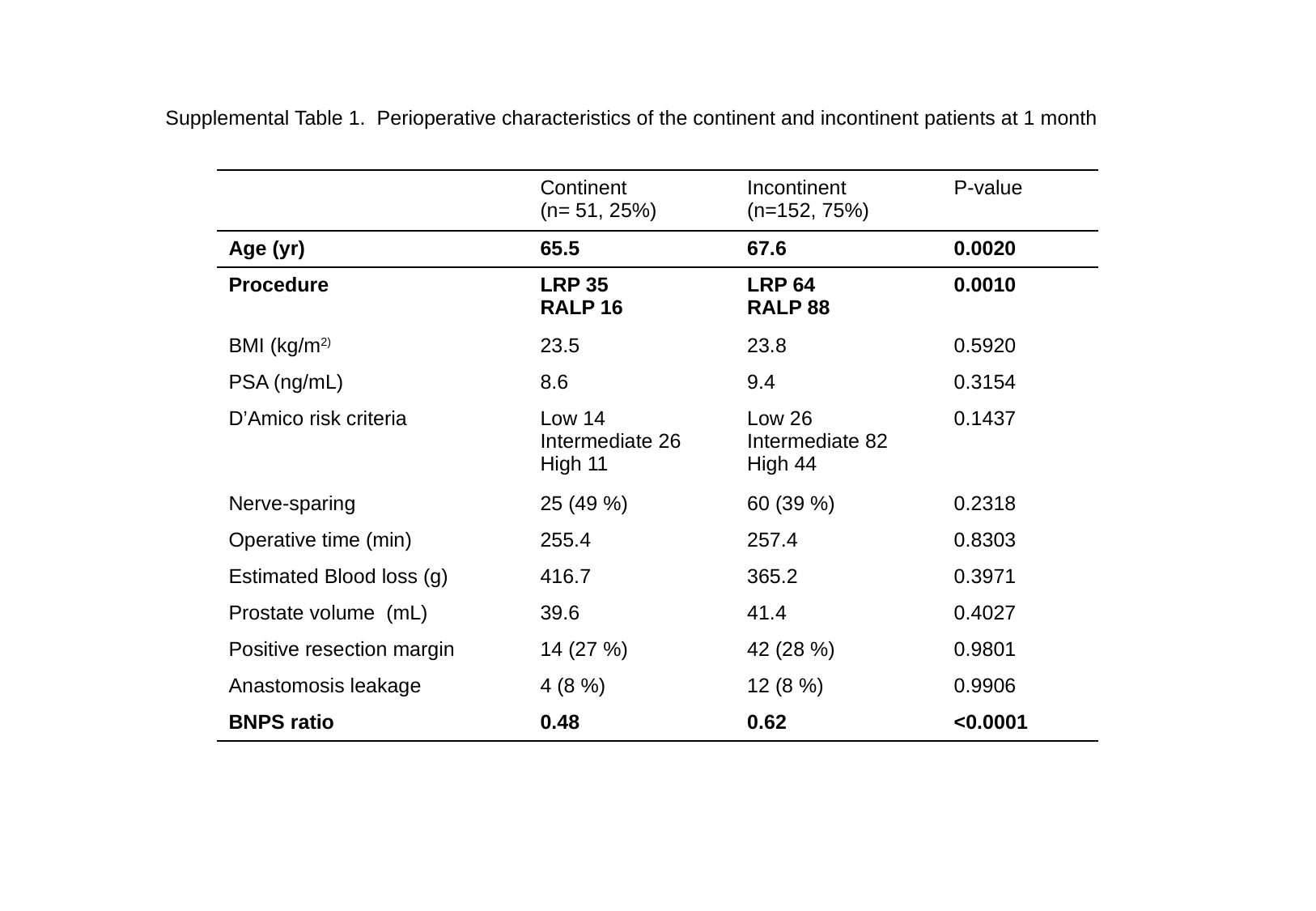

Supplemental Table 1. Perioperative characteristics of the continent and incontinent patients at 1 month
| | Continent (n= 51, 25%) | Incontinent (n=152, 75%) | P-value |
| --- | --- | --- | --- |
| Age (yr) | 65.5 | 67.6 | 0.0020 |
| Procedure | LRP 35 RALP 16 | LRP 64 RALP 88 | 0.0010 |
| BMI (kg/m2) | 23.5 | 23.8 | 0.5920 |
| PSA (ng/mL) | 8.6 | 9.4 | 0.3154 |
| D’Amico risk criteria | Low 14 Intermediate 26 High 11 | Low 26 Intermediate 82 High 44 | 0.1437 |
| Nerve-sparing | 25 (49 %) | 60 (39 %) | 0.2318 |
| Operative time (min) | 255.4 | 257.4 | 0.8303 |
| Estimated Blood loss (g) | 416.7 | 365.2 | 0.3971 |
| Prostate volume (mL) | 39.6 | 41.4 | 0.4027 |
| Positive resection margin | 14 (27 %) | 42 (28 %) | 0.9801 |
| Anastomosis leakage | 4 (8 %) | 12 (8 %) | 0.9906 |
| BNPS ratio | 0.48 | 0.62 | <0.0001 |

## Slide 2
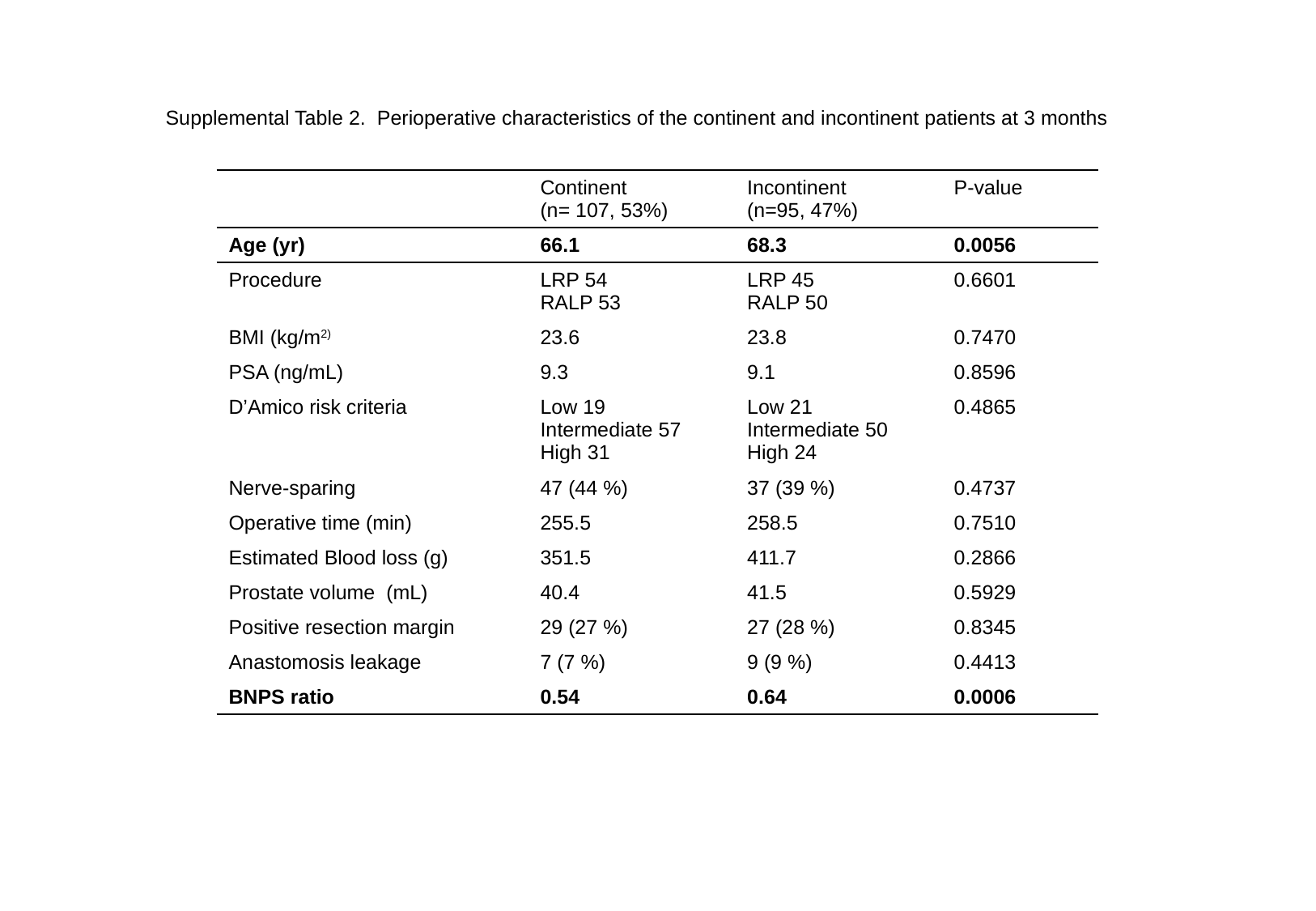

Supplemental Table 2. Perioperative characteristics of the continent and incontinent patients at 3 months
| | Continent (n= 107, 53%) | Incontinent (n=95, 47%) | P-value |
| --- | --- | --- | --- |
| Age (yr) | 66.1 | 68.3 | 0.0056 |
| Procedure | LRP 54 RALP 53 | LRP 45 RALP 50 | 0.6601 |
| BMI (kg/m2) | 23.6 | 23.8 | 0.7470 |
| PSA (ng/mL) | 9.3 | 9.1 | 0.8596 |
| D’Amico risk criteria | Low 19 Intermediate 57 High 31 | Low 21 Intermediate 50 High 24 | 0.4865 |
| Nerve-sparing | 47 (44 %) | 37 (39 %) | 0.4737 |
| Operative time (min) | 255.5 | 258.5 | 0.7510 |
| Estimated Blood loss (g) | 351.5 | 411.7 | 0.2866 |
| Prostate volume (mL) | 40.4 | 41.5 | 0.5929 |
| Positive resection margin | 29 (27 %) | 27 (28 %) | 0.8345 |
| Anastomosis leakage | 7 (7 %) | 9 (9 %) | 0.4413 |
| BNPS ratio | 0.54 | 0.64 | 0.0006 |

## Slide 3
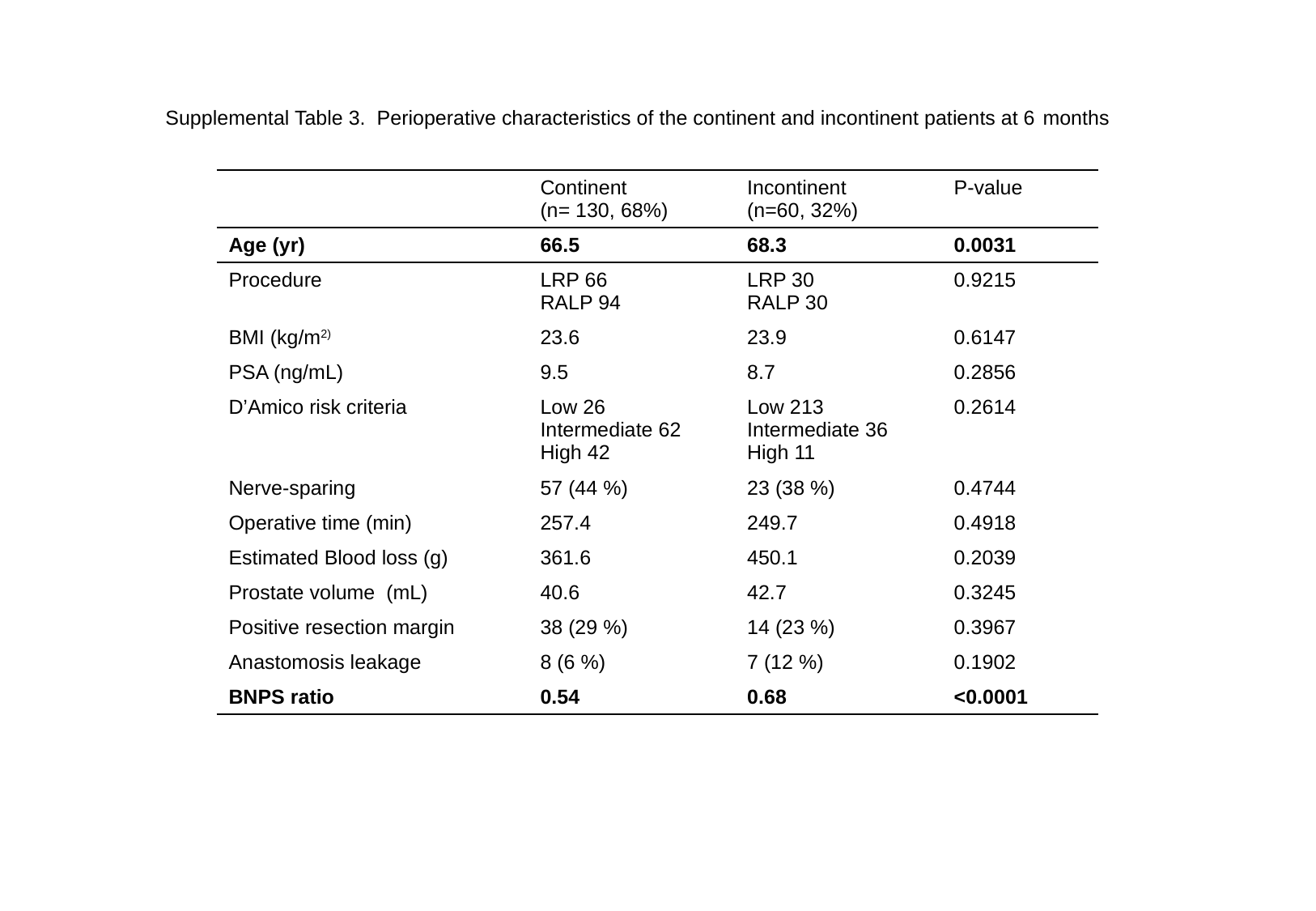

Supplemental Table 3. Perioperative characteristics of the continent and incontinent patients at 6 months
| | Continent (n= 130, 68%) | Incontinent (n=60, 32%) | P-value |
| --- | --- | --- | --- |
| Age (yr) | 66.5 | 68.3 | 0.0031 |
| Procedure | LRP 66 RALP 94 | LRP 30 RALP 30 | 0.9215 |
| BMI (kg/m2) | 23.6 | 23.9 | 0.6147 |
| PSA (ng/mL) | 9.5 | 8.7 | 0.2856 |
| D’Amico risk criteria | Low 26 Intermediate 62 High 42 | Low 213 Intermediate 36 High 11 | 0.2614 |
| Nerve-sparing | 57 (44 %) | 23 (38 %) | 0.4744 |
| Operative time (min) | 257.4 | 249.7 | 0.4918 |
| Estimated Blood loss (g) | 361.6 | 450.1 | 0.2039 |
| Prostate volume (mL) | 40.6 | 42.7 | 0.3245 |
| Positive resection margin | 38 (29 %) | 14 (23 %) | 0.3967 |
| Anastomosis leakage | 8 (6 %) | 7 (12 %) | 0.1902 |
| BNPS ratio | 0.54 | 0.68 | <0.0001 |

## Slide 4
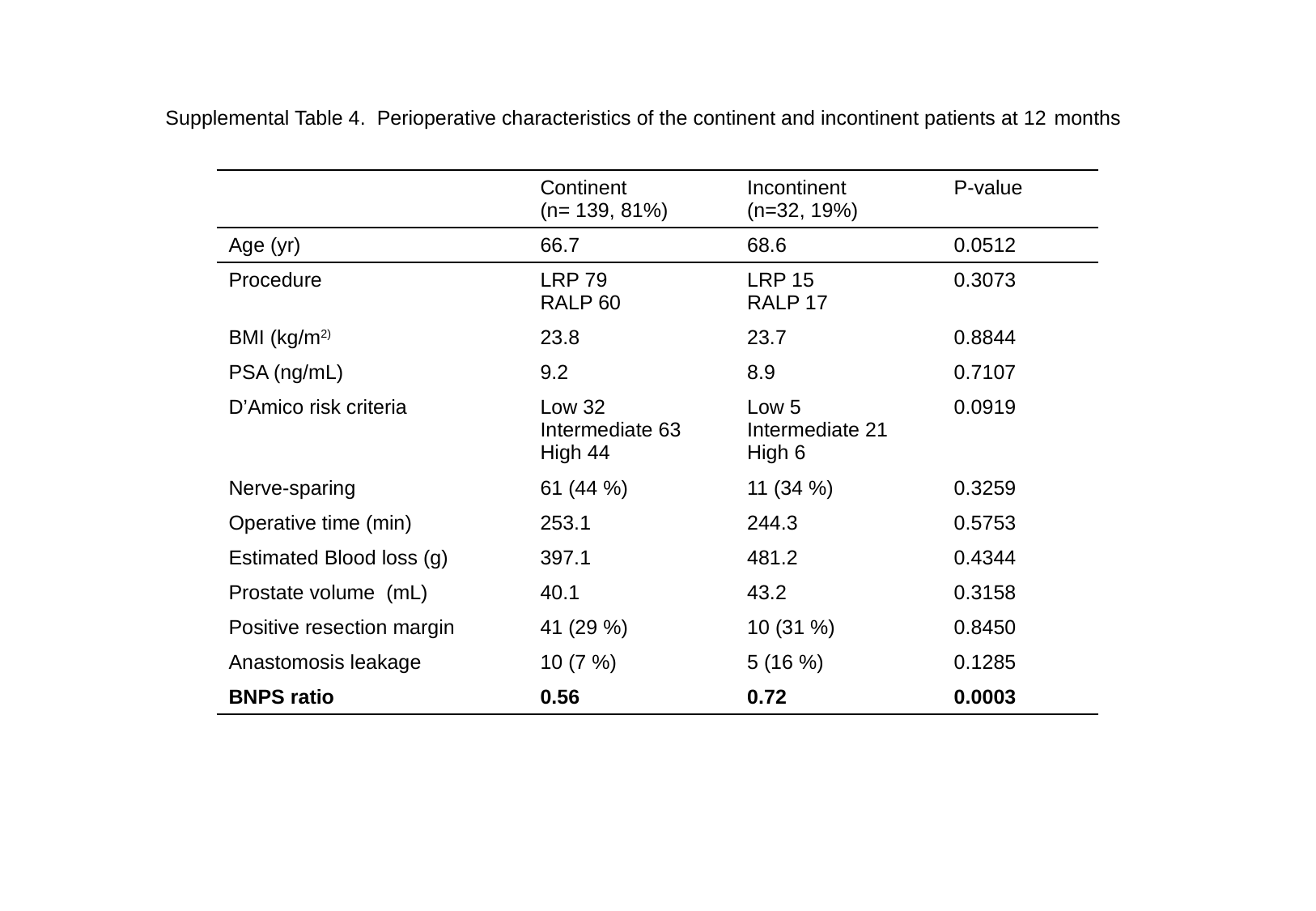

Supplemental Table 4. Perioperative characteristics of the continent and incontinent patients at 12 months
| | Continent (n= 139, 81%) | Incontinent (n=32, 19%) | P-value |
| --- | --- | --- | --- |
| Age (yr) | 66.7 | 68.6 | 0.0512 |
| Procedure | LRP 79 RALP 60 | LRP 15 RALP 17 | 0.3073 |
| BMI (kg/m2) | 23.8 | 23.7 | 0.8844 |
| PSA (ng/mL) | 9.2 | 8.9 | 0.7107 |
| D’Amico risk criteria | Low 32 Intermediate 63 High 44 | Low 5 Intermediate 21 High 6 | 0.0919 |
| Nerve-sparing | 61 (44 %) | 11 (34 %) | 0.3259 |
| Operative time (min) | 253.1 | 244.3 | 0.5753 |
| Estimated Blood loss (g) | 397.1 | 481.2 | 0.4344 |
| Prostate volume (mL) | 40.1 | 43.2 | 0.3158 |
| Positive resection margin | 41 (29 %) | 10 (31 %) | 0.8450 |
| Anastomosis leakage | 10 (7 %) | 5 (16 %) | 0.1285 |
| BNPS ratio | 0.56 | 0.72 | 0.0003 |

## Slide 5
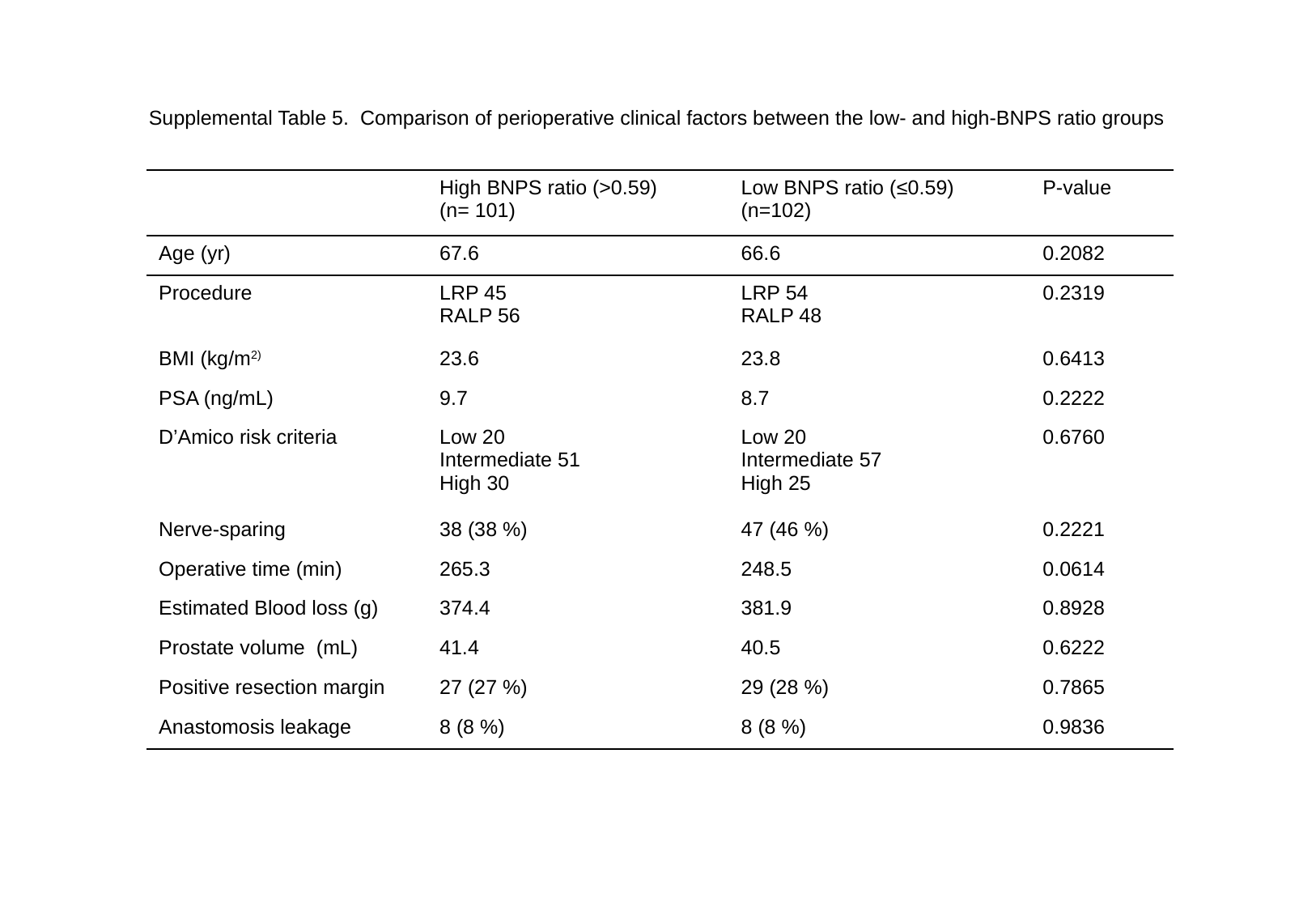

Supplemental Table 5. Comparison of perioperative clinical factors between the low- and high-BNPS ratio groups
| | High BNPS ratio (>0.59) (n= 101) | Low BNPS ratio (≤0.59) (n=102) | P-value |
| --- | --- | --- | --- |
| Age (yr) | 67.6 | 66.6 | 0.2082 |
| Procedure | LRP 45 RALP 56 | LRP 54 RALP 48 | 0.2319 |
| BMI (kg/m2) | 23.6 | 23.8 | 0.6413 |
| PSA (ng/mL) | 9.7 | 8.7 | 0.2222 |
| D’Amico risk criteria | Low 20 Intermediate 51 High 30 | Low 20 Intermediate 57 High 25 | 0.6760 |
| Nerve-sparing | 38 (38 %) | 47 (46 %) | 0.2221 |
| Operative time (min) | 265.3 | 248.5 | 0.0614 |
| Estimated Blood loss (g) | 374.4 | 381.9 | 0.8928 |
| Prostate volume (mL) | 41.4 | 40.5 | 0.6222 |
| Positive resection margin | 27 (27 %) | 29 (28 %) | 0.7865 |
| Anastomosis leakage | 8 (8 %) | 8 (8 %) | 0.9836 |
